# Assessment of Trends in Second Primary Cancers in Patients With Metastatic Melanoma From 2005 to 2016

**DOI:** 10.1001/jamanetworkopen.2020.28627

**Published:** 2020-12-09

**Authors:** Weiye Deng, Yifan Wang, Xiangyu Liu, Jieqiong Liu, Liang Wang, Zhaogang Yang, Mingming Yang, Yi An, Chad Tang, Nina N. Sanford, Betty Y. S. Kim, Wen Jiang

**Affiliations:** 1 Department of Radiation Oncology, University of Texas Southwestern Medical Center, Dallas; 2 Department of Biostatistics and Data Science, School of Public Health, the University of Texas Health Science Center, Houston; 3 Guangdong Provincial Key Laboratory of Malignant Tumor Epigenetics and Gene Regulation, Sun Yat-sen Memorial Hospital, Sun Yat-sen University, Guangzhou, China; 4 Breast Tumor Center, Sun Yat-sen Memorial Hospital, Sun Yat-sen University, Guangzhou, China; 5 Department of Radiation Oncology, University of Texas MD Anderson Cancer Center, Houston; 6 Department of Therapeutic Radiology, Yale School of Medicine, New Haven, Connecticut; 7 Department of Neurosurgery, University of Texas MD Anderson Cancer Center, Houston

## Abstract

**Importance:**

To date, the risk of developing second primary cancers (SPCs) after the first primary melanoma has not been studied in the era of immune checkpoint inhibitors (ICIs).

**Objective:**

To assess differences in the risk of SPCs in patients with primary melanoma before (2005-2010) and after (2011-2016) the introduction and approval of ICIs.

**Design, Setting, and Participants:**

Population-based cohort study using the Surveillance, Epidemiology, and End Results database from January 2005 to December 2016 of patients diagnosed with metastatic melanoma. Data were analyzed from January 4 to June 30, 2020.

**Exposures:**

Receipt of immunotherapy or other anticancer agents.

**Main Outcomes and Measures:**

The primary outcome was the development of second primary cancers in patients with melanoma. Standardized incidence ratios (SIRs) were calculated for the development of SPCs before and after the introduction of ICIs.

**Results:**

Among 5016 patients with diagnosed metastatic melanoma, 2888 (58%) were younger than 65 years at the time of diagnosis, and 3441 (69%) were male. From 2005 to 2010, SIRs were 3.24 (95% CI, 0.08-18.04) for small intestine cancer, 1.93 (95% CI, 1.14-3.05) for lung and bronchus cancer, 2.77 (95% CI, 1.02-6.03) for kidney cancer, and 7.29 (95% CI, 2.93-15.02) for myeloma. From 2011 to 2016, SIRs were 9.23 (95% CI, 1.12-33.35) for small intestine cancer, 1.54 (95% CI, 0.71-2.93) for lung and bronchus cancer, 2.66 (95% CI, 0.73-6.82) for kidney cancer, and 5.90 (95% CI, 1.61-15.10) for myeloma. The overall risk of developing SPCs in individuals who survived the first primary melanoma was 65% higher (SIR, 1.65; 95% CI, 1.35-2.00) in the pre-ICIs period and 98% higher (SIR, 1.98; 95% CI, 1.57-2.45) in the post-ICIs period than the overall cancer incidence rate in the general population.

**Conclusions and Relevance:**

In this study, an increase in the overall risk of second primary cancers after melanoma after the introduction of immune checkpoint inhibitors was observed. The pattern of SPCs has been altered in the era of systemic therapy. Close monitoring and screening for SPCs may be warranted in patients with metastatic melanoma.

## Key Points

**Question:**

When compared with prior therapies, are immune checkpoint inhibitors for metastatic melanoma associated with development of second primary cancers?

**Findings:**

In this cohort study of 5016 patients with metastatic melanoma between 2005 and 2016, the overall risk of second primary cancers increased after the introduction of immune checkpoint inhibitors. Risks for cancer of the small intestine and myeloma were higher than before the era of immune checkpoint inhibitors.

**Meaning:**

In this study, a change in the incidence of second primary cancers was found; screening for these cancers may be warranted in patients treated with immune checkpoint inhibitors for metastatic melanoma.

## Teaser

This cohort study assesses the risk of second primary cancers in patients with primary melanoma before (2005-2010) and after (2011-2016) the introduction and approval of immune checkpoint inhibitors.

## Introduction

Melanoma is one of the most common cancer types and one of the few cancers with increasing incidence in the US.^1^ Although treatment options for patients with melanoma, including chemotherapy, surgery, and radiation, have evolved, achieving optimum treatment outcomes continues to be challenging.^2^ This evolution is especially true for metastatic melanoma because it is usually highly resistant to the standard of care.^3^ The introduction of immune checkpoint inhibitors (ICIs) has substantially improved clinical outcomes in patients with advanced-stage cancers. For example, the 5-year overall survival rate for metastatic melanoma increased from approximately 9% to 18% with ipilimumab.^4^ After the approval of ipilimumab in 2011, other ICIs including nivolumab and pembrolizumab were approved by the US Food and Drug Administration (FDA) for treatment of metastatic melanoma. Because patients with advanced-stage cancers are living longer, long-term treatment and disease-related sequelae are becoming increasingly common on follow-up. One of the most life-threatening sequelae, second primary cancers (SPCs), is often overlooked in the era of immunotherapy.^5,6^ However, to our knowledge, the risk of SPCs among patients with metastatic melanoma has not been assessed after ICIs were introduced.

We used the Surveillance, Epidemiology, and End Results (SEER) database to assess differences in the risk of SPCs in patients with primary melanoma before and after the FDA approved ICIs. We also analyzed changes in the incidence of SPCs with the increased adoption of ICIs as part of the standard of care. Our study hypothesis is that the pattern of SPCs that develop in patients with melanoma has altered in recent years.

## Methods

### Data Source and Case Definition

Our hypothesis is based on the assumption that patients who have been diagnosed with metastatic melanoma since 2011 are more likely to have received immunotherapy. We tested our hypothesis by using SEER data to evaluate the standardized incidence ratios (SIRs) for second primary cancers.

We studied 5016 patients diagnosed with metastatic melanoma between 2005 and 2016 in the SEER 18 database. The SEER registry is a database of 18 cancer registries from across the United States that represents approximately 28% of the US population.^7^ The SEER database is periodically updated with patient information, including patient demographic characteristics, tumor characteristics, initial treatment modality, diagnosis date, and death date. This study followed the Strengthening the Reporting of Observational Studies in Epidemiology (STROBE) reporting guideline for cohort studies. Data were analyzed from January 4 to June 30, 2020. This cohort study was exempt from institutional review board approval and informed consent by the Institutional Review Board at the University of Texas Southwestern Medical Center because study participants were ascertained through a deidentified and publicly available database.

We separated the selected cohort into 2 groups by time period: pre-ICIs (2005-2010) and post-ICIs (2011-2016). Patients were included at least 2 months after their initial melanoma diagnosis and were followed up through December 31, 2016. To minimize detection bias, we excluded synchronous cancers (diagnosed within 2 months of the index malignancy)^8^ and did not consider third or more subsequent primary cancers. The histology of melanoma was defined according to the *International Classification of Diseases for Oncology, Third Revision*, as follows: superficial spreading melanoma (category 8743), nodular melanoma (8721), lentigo maligna melanoma (8742), acral lentiginous melanoma (8744), and melanoma, not otherwise specified (8720), or other (codes 8722, 8723, 8730, 8740, 8741, 8745, 8761, and 8770-8774).^9^

### Statistical Analysis

The primary outcomes were overall survival and development of SPCs in patients with metastatic melanoma. Patients with metastatic melanoma were followed up to the date they developed SPCs, date of death, date of the last follow-up, or end of the study period. We used univariate and multivariate Cox proportional hazards regression models to identify possible risk factors for SPC development. Standardized incidence ratios were calculated as a measure of the relative risk of SPCs by using SEER*Stat version 8.3.6^10^ and by dividing the observed number of cases of second primary cancer by the expected number of cases in the general population after matching by sex, age group, and calendar year.^10^ We used SIR to estimate the risk that survivors of metastatic melanoma will develop a second primary malignancy relative to the incidence of cancer among the general population.^11^ Two-sided 95% CIs for SIRs were calculated assuming a Poisson distribution for the observed number of subsequent SPCs. We examined SIRs for 10 common types of second cancers, in combination and individually. We plotted the cumulative incidence of all-cause mortality after melanoma diagnosis, stratified by era (pre- and post-ICIs) and by sex. Because death could be considered a competing event for SPCs during follow-up, we also used competing-risk cumulative incidence curves to estimate the cumulative incidence of SPCs.^12,13^ Statistical analyses were performed with SAS software version 9.4 (SAS Institute Inc), and R version 3.6.1 (The R Project for Statistical Computing). Tests were 2-sided and conducted at α = .05.

## Results

A total of 5016 patients in the SEER 18 database were diagnosed with metastatic melanoma from 2005 to 2016. Of these, 46% were diagnosed in the pre-ICIs era, and 54% were in the post-ICIs era. The median observed survival time of patients with metastatic melanoma was 11 months (range, 2-143 months) in the pre-ICIs era and 10 months (range, 2-71 months) in the post-ICIs era. Among them, 401 of 2315 patients (17%) and 79 of 2701 patients (3%) in the pre-ICIs and post-ICIs eras had 5-year follow-up. The end date of SPC diagnosis is December 2016. A total of 190 (4%) patients developed SPCs: 107 in the pre-ICIs era and 83 in the post-ICIs era (Table 1). Less than half of patients (42%) were age 65 years or older. Most patients (96.3%) were White. In univariate Cox proportional hazards regression model analysis, sites of initial melanoma diagnosis, age, histology, and treatment were identified as risk factors for the development of SPCs in patients with melanoma (eTable 1 in the Supplement). In multivariate analysis, only age emerged as an independent risk factor (risk ratio, 1.02; 95% CI, 1.01-1.03, *P* < .001).

**Table 1.** Demographic and Disease Characteristics of Patients in SEER, 2005-2010 and 2011-2016

Characteristic	No. (%)	
	2005-2010 (n = 2315)	2011-2016 (n = 2701)
Sex		
Male	1567 (67.7)	1874 (69.4)
Female	748 (32.3)	827 (30.6)
Race^a^		
White	2220 (96.2)	2594 (96.3)
Non-White	88 (3.8)	99 (3.7)
Age at diagnosis of first melanoma, y		
<65	1397 (60.3)	1491 (55.2)
≥65	918 (39.7)	1210 (44.8)
Site of first melanoma		
Head and neck	237 (10.2)	281 (10.4)
Trunk	355 (15.3)	356 (13.2)
Upper limbs/shoulder	158 (6.8)	166 (6.1)
Lower limbs/hip	202 (8.7)	213 (7.9)
Other^b^	1363 (58.9)	1685 (62.4)
Histologic finding		
SSM	81 (3.5)	89 (3.3)
NM	212 (9.2)	252 (9.3)
LMM	2 (0.1)	18 (0.7)
ALM	24 (1.0)	22 (0.8)
Melanoma, NOS	1866 (80.6)	2165 (80.2)
Other^c^	130 (5.6)	155 (5.7)
**Initial treatment**		
Surgical treatment		
Yes	1477 (63.8)	1876 (69.5)
No	838 (36.2)	825 (30.5)
Radiotherapy		
Yes	905 (39.1)	819 (30.3)
No	1410 (60.9)	1882 (69.7)
Chemotherapy		
Yes	913 (39.4)	819 (30.3)
No	1402 (60.6)	1882 (69.7)
SPC		
Yes	107 (4.6)	83 (3.1)
No	2208 (95.4)	2618 (96.9)

Abbreviations: ALM, acral lentiginous melanoma; LMM, lentigo maligna melanoma; NM, nodular melanoma; NOS, not otherwise specified; SEER, Surveillance, Epidemiology, and End Results; SPC, second primary cancer; SSM, superficial spreading melanoma.

^a^ Race data were available for 5001 patients.

^b^ Includes overlapping areas of skin and not otherwise specified.

^c^ Other includes balloon cell, amelanotic, desmoplastic, mucosal lentiginous, mixed epithelioid/spindle cell, epithelioid, and spindle cell melanomas.

**Figure 1.**
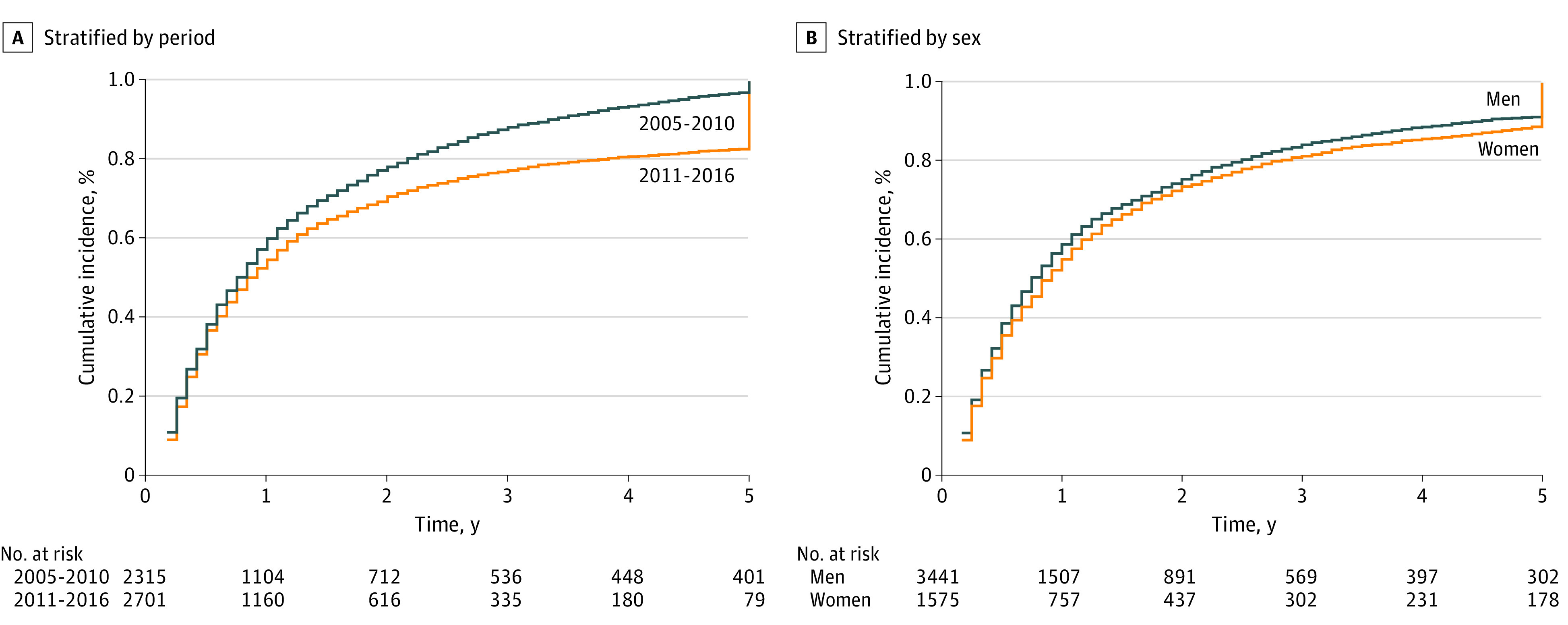
Five-Year Cumulative Incidence Function of All-Cause Mortality A, Stratified by 2005-2010 and 2011-2016 time periods. B, Stratified by sex. Years of observation were compiled from the date of first primary melanoma diagnosis to the date of death or last follow-up.

**Figure 2.**
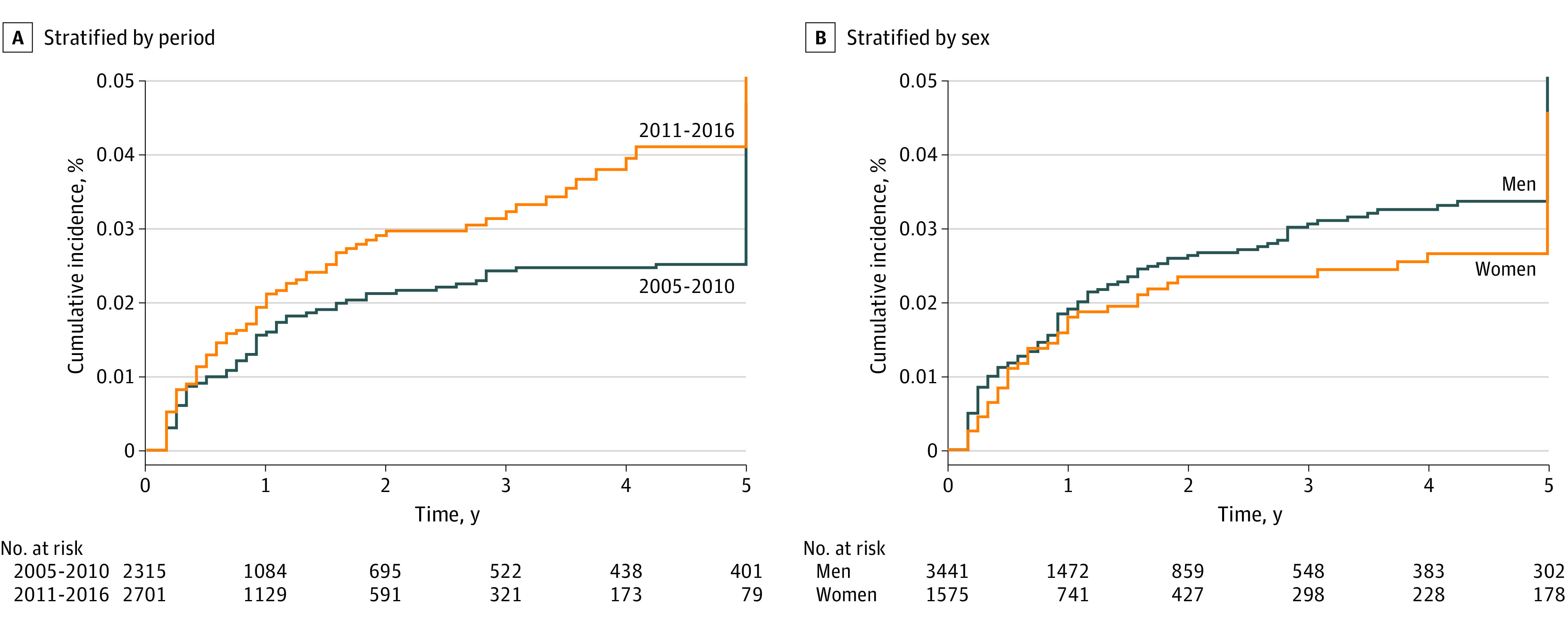
Five-Year Cumulative Incidence of Second Primary Cancers With Death as a Competing Risk A, Stratified by 2005-2010 and 2011-2016 time periods. B, Stratified by sex. Years of observation were compiled from the date of the first primary melanoma diagnosis to the date of diagnosis of second primary cancers.

The 5-year cumulative incidence of all-cause mortality was 0.82 (95% CI, 0.81-0.84) in the pre-ICIs group and 0.76 (95% CI, 0.73-0.78) in the post-ICIs group (*P* < .001) (Figure 1A). The estimated cumulative incidence of SPCs in the competing-risk model at the 5-year mark was 4.7% in the pre-ICIs era and 5.3% in the post-ICIs era (Figure 2A). The periods were not statistically significantly associated with SPCs, though we observed increasing risk from the pre- to the post-ICIs period (*P* = .08). The 5-year cumulative incidence of all-cause mortality was 0.81 (95% CI, 0.79-0.82) in men and 0.77 (95% CI, 0.75-0.79) in women (*P* = .003) (Figure 1B). The 5-year cumulative incidence of SPCs did not differ significantly between men (5.8%) and women (4.6%) upon competing risk analysis (*P* = .16) (Figure 2B).

The overall risk of developing SPCs in survivors of the first primary melanoma was 65% higher (SIR, 1.65; 95% CI, 1.35-2.00) in the pre-ICIs period and 98% higher (SIR, 1.98; 95% CI, 1.57-2.45) in the post-ICIs period than the overall cancer incidence rate in the general population (Table 2; eTable 2 in the Supplement). When we excluded the effect of melanoma, the overall risk for all sites was 1.26 (95% CI, 0.99-1.57) in the pre-ICIs period and 1.42 (95% CI, 1.08-1.85) in the post-ICIs period. In the pre-ICIs period, SIRs were 3.24 (95% CI, 0.08-18.04) for small intestine cancer, 1.93 (95% CI, 1.14-3.05) for lung and bronchus cancer, 2.77 (95% CI, 1.02-6.03) for kidney cancer, and 7.29 (95% CI, 2.93-15.02) for myeloma; the excess risk of SPCs was primarily due to myeloma and chronic lymphocytic leukemia. In contrast, in the post-ICIs period, SIRs were 9.23 (95% CI, 1.12-33.35) for small intestine cancer, 1.54 (95% CI, 0.71-2.93) for lung and bronchus cancer, 2.66 (95% CI, 0.73-6.82) for kidney cancer, and 5.90 (95% CI, 1.61-15.10) for myeloma; the excess risk of SPCs was primarily due to intestinal cancers and myeloma (Table 2; eTable 2 in the Supplement). When the analysis was stratified by sex, the overall risk of developing SPCs in survivors of first primary melanoma was similar in men (SIR, 1.80; 95% CI, 1.51-2.12) and women (SIR, 1.73; 95% CI, 1.30-2.27). When we excluded patients with secondary melanoma, the overall risk of developing SPCs was statistically significantly higher in men (SIR, 1.34; 95% CI, 1.08-1.63) than in women (SIR, 1.29; 95% CI, 0.91-1.77). The excess risk of SPCs was mainly due to myeloma in both groups (Table 3; eTable 3 in the Supplement).

**Table 2.** SIRs of Selected SPC Sites After Initial Melanoma Diagnosis in 2005-2010 and 2011-2016

SPC site	2005-2010		2011-2016	
	Observed No.	SIR (95% CI)	Observed No.	SIR (95% CI)
All sites	107	1.65 (1.35-2.00)^a^	83	1.98 (1.57-2.45)^a^
All sites excluding melanoma	77	1.26 (0.99-1.57)	56	1.42 (1.08-1.85)^a^
All solid tumors	89	1.56 (1.25-1.92)^a^	73	1.98 (1.55-2.49)^a^
Small intestine	1	3.24 (0.08-18.04)	2	9.23 (1.12-33.35)^a^
Colon and rectum	4	0.66 (0.18-1.70)	4	1.09 (0.30-2.78)
Lung and bronchus	18	1.93 (1.14-3.05)^a^	9	1.54 (0.71-2.93)
Melanoma of the skin	30	8.45 (5.70-12.07)^a^	27	10.11 (6.66-14.71)^a^
Female breast	6	1.13 (0.41-2.45)	5	1.55 (0.50-3.61)
Prostate	8	0.63 (0.27-1.24)	7	0.92 (0.37-1.89)
Kidney	6	2.77 (1.02-6.03)^a^	4	2.66 (0.73-6.82)
Thyroid	2	2.13 (0.26-7.70)	0	0
Non-Hodgkin lymphoma	4	1.39 (0.38-3.57)	3	1.55 (0.32-4.52)
Myeloma	7	7.29 (2.93-15.02)^a^	4	5.90 (1.61-15.10)^a^
Chronic lymphocytic leukemia	4	4.57 (1.24-11.70)^a^	0	0

Abbreviations: SIR, standardized incidence ratio; SPC, second primary cancer.

^a^ Statistically significant value (*P* < .05).

**Table 3.** SIRs of Selected SPC Sites After Initial Melanoma Diagnosis in Male and Female Patients

SPC site	Male		Female	
	Observed No.	SIR (95% CI)	Observed No.	SIR (95% CI)
All sites	138	1.80 (1.51-2.12)^a^	52	1.73 (1.30-2.27)^a^
All sites excluding melanoma	96	1.34 (1.08-1.63)^a^	37	1.29 (0.91-1.77)
All solid tumors	115	1.71 (1.41-2.05)^a^	47	1.76 (1.30-2.34)^a^
Small intestine	2	5.26 (0.64-19.01)	1	6.88 (0.17-38.31)
Colon and rectum	5	0.73 (0.24-1.70)	3	1.05 (0.22-3.07)
Lung and bronchus	22	2.04 (1.28-3.08)^a^	5	1.14 (0.37-2.67)
Melanoma of the skin	42	8.42 (6.07-11.39)^a^	15	12.16 (6.80-20.05)^a^
Female breast	0	0	11	1.29 (0.64-2.30)
Prostate	15	0.74 (0.41-1.22)	0	0
Kidney	6	2.04 (0.75-4.45)	4	5.47 (1.49-14.02)^a^
Thyroid	2	2.62 (0.32-9.47)	0	0
Non-Hodgkin lymphoma	7	1.99 (0.80-4.11)	0	0
Myeloma	8	6.49 (2.80-12.80)^a^	3	7.38 (1.52-21.57)^a^
Chronic lymphocytic leukemia	3	2.63 (0.54-7.70)	1	3.06 (0.08-17.05)

Abbreviations: SIR, standardized incidence ratio; SPC, second primary cancer.

^a^ Statistically significant value (*P* < .05).

## Discussion

To our knowledge, this is the first population-based study that describes SPCs in patients with metastatic melanoma before and after the introduction of ICIs in the United States. We observed a higher SIR for SPCs in the post-ICIs era. We also observed higher SPC risk when we used death as a competing factor on a 5-year basis and stratified by the 2 time periods. We found that SPCs in patients with metastatic melanoma shifted from hematological cancers in the pre-ICIs era to a mix of cancer of the small intestine and myeloma in the post-ICIs era. Therefore, patients might need continuous surveillance not only for melanoma recurrence but also for new primary melanomas and other cancers. We also observed a slightly higher risk of SPCs in men than in women, though a study conducted from 1992 to 2006 by Balamurugan et al.^14^ showed a slightly higher risk of SPCs in women than in men. A systematic meta-analysis by Caini et al.^5^ evaluated 23 melanoma studies with more than 350 000 patients and found that the overall SIR for SPCs was 1.57 (95% CI, 1.29-1.90). These melanoma cases were recruited from 1943 to 2007, and the SIRs for SPCs in sites including breast in women (SIR, 1.14; 95% CI, 1.07-1.22) and non-Hodgkin lymphoma (SIR, 1.37; 95% CI, 1.22-1.54) were similar to the results we obtained for the pre-ICIs era.^5^ However, kidney (SIR, 1.34; 95% CI, 1.23-1.45), prostate (SIR, 1.25; 95% CI, 1.13-1.37), and colon (SIR, 1.12; 95% CI, 1.00-1.25) results in this meta-analysis were different from those of the current study, which might be attributed to changing patterns of SPCs in different time periods. Immune checkpoint inhibitor treatment improves survival in some patients, but it also causes immune-related adverse events (irAEs) that can affect healthy tissues in the body.^15,16^ Our study provides insight into the changing pattern of SPCs after the introduction of ICIs and indicates the need for long-term follow-up and monitoring of SPCs in this patient population.

Ipilimumab was approved by the FDA for treating metastatic melanoma in 2011,^17^ and numerous targeted therapies have been approved by the FDA since then. Immune checkpoint inhibitors and targeted therapies have emerged as key components of metastatic melanoma treatment in recent years.^18,19^ Immune checkpoint inhibitors might present a more severe toxicity profile with organ-specific irAEs than traditional treatments.^20^ Among these adverse events, gastrointestinal toxicities are some of the most common irAEs.^21^ Our study observed an increased risk of SPCs for cancer of the small intestine. Although such observations might be correlative rather than causative, it is possible that the irAEs affect the development and progression of SPCs. The exact relationship between gastrointestinal irAEs and SPCs needs further investigation. Most previous studies of irAEs have focused on the short-term toxicity of ICIs with a duration of weeks to months, but the long-term effect of ICIs has not been broadly investigated. Furthermore, using ICIs has dramatically improved melanoma outcomes. With patients now surviving longer after treatment, it is becoming increasingly important and clinically relevant to better understand the patterns of SPCs in the ICIs era to promote the best quality of life of patients. Although better rates of incidental detection with more sensitive imaging tools and more frequent use of ultrasound may explain the increase in incidence, we also expect more sensitive imaging technology to detect other solid tumors, which we did not observe here. Very importantly, we cannot determine whether the change in the pattern of SPCs is caused by ICIs; future studies are warranted to further elucidate the relationship between ICIs and SPCs, particularly those focused on the risk of subsequent cancer after immunotherapy.

Because SPCs are rare and usually emerge long after treatment, the SEER database is an ideal tool for evaluating them, because it provides a large nationwide sample with long-term follow-up. The results of analyzing these data can represent real-world practice patterns, trends, and outcomes that cannot be ascertained from a randomized clinical trial.^22^ The SEER database also enables the evaluation of SPC risk according to a variety of relevant variables and yields results that may be generalizable to the entire population. By controlling the treatment and demographic variables the database provides, we can minimize the potential for bias.

### Limitations

This study has limitations. The SEER database lacks detailed information on cancer risk factors, including socioeconomic status, genetic variants, and behavior factors.^23^ Other factors that might be associated with the changes in incidence of SPCs but that are not in the SEER database include differences over time in disease severity at diagnosis, differences in exposure history to environmental carcinogens, differences in overall health and comorbidities, differences in availability of and barriers to medical care, and differences in other aspects of medical care than immunotherapy. Another drawback is that treatment data at the individual level are not available; thus, we used the year of metastatic melanoma diagnosis as a surrogate for the type of treatment. The increased risk of some SPCs could be associated with ICI, as this is the main change to melanoma care that could be associated with secondary malignancy. It is possible that some patients in the pre-ICIs group received ICIs at a later date or that a portion of the patients in the post-ICIs group did not receive ICIs. Furthermore, the follow-up periods for the pre- and post-ICIs groups are different. Indeed, risk is expected to accumulate with a longer follow-up. However, the fact that changes in the pattern and incidence of SPCs in the post-ICI era can be observed even with a shorter follow-up period emphasizes the possible clinical relevance of the relationship between ICIs and SPCs.

Despite these limitations, to our knowledge, this is the first study that evaluates SPCs after metastatic melanoma before and after the approval of ICIs by the FDA. More clinical and mechanistic studies are warranted.

## Conclusions

In this cohort study, we found a changing incidence of second primary cancers after metastatic melanoma that shifted in the era of systemic therapy from hematological cancers to a mix of cancer of the small intestine and myeloma. As ICIs continue to gain approval as part of the standard-of-care treatment for multiple cancers, our findings highlight the importance of tailored monitoring approaches for patients treated with immunotherapy and further identifying potential risk factors associated with subsequent cancer development. Vigilant individualized monitoring and screening for these cancers are warranted.

## Notes

Supplement. **eTable 1.** Univariate Analysis and Multivariate Analysis for Risk of Developing SPM Over Time After Index Cancer **eTable 2.** SIRs of all SPM Sites After Initial Melanoma Diagnosis Between 2005-2010 and 2011-2016 Time Periods **eTable 3.** SIRs of Selected SPMs Sites After Initial Melanoma Diagnosis Between Males and Females
