# Supplementary material for: Assessment of Trends in Second Primary Cancers in Patients With Metastatic Melanoma From 2005 to 2016
Source: JAMA Netw Open. 2020 Dec 9;3(12):e2028627. doi: 10.1001/jamanetworkopen.2020.28627 (PMC7726633; doi:10.1001/jamanetworkopen.2020.28627)
Supplement: Supplement. — eTable 1. Univariate Analysis and Multivariate Analysis for Risk of Developing SPM Over Time After Index Cancer eTable 2. SIRs of all SPM Sites After Initial Melanoma Diagnosis Between 2005-2010 and 2011-2016 Time Periods eTable 3. SIRs of Selected SPMs Sites After Initial Melanoma Diagnosis Between Males and Females [file jamanetwopen-e2028627-s001.pdf]

Supplemental Online Content

Deng W, Wang Y, Liu X, et al. Assessment of trends in second primary cancers in patients with metastatic melanoma from 2005 to 2016. *JAMA Netw Open.* 2020;3(12):e2028627. doi:10.1001/jamanetworkopen.2020.28627

eTable 1. Univariate Analysis and Multivariate Analysis for Risk of Developing SPM Over Time After Index Cancer

eTable 2. SIRs of all SPM Sites After Initial Melanoma Diagnosis Between 2005-2010 and 2011-2016 Time Periods

eTable 3. SIRs of Selected SPMs Sites After Initial Melanoma Diagnosis Between Males and Females

This supplemental material has been provided by the authors to give readers additional information about their work.

© 2020 Deng W et al. *JAMA Network Open*.

eTable 1. Univariate analysis and multivariate analysis for risk of developing SPM over time after index cancer.

Parameters	Risk ratio	95% CI[a]	*P*	Risk ratio	95% CI[a]	*P*
Sex						
Male	Reference			-		
Female	0.74	0.54-1.02	.06			
race						
White	Reference			-		
Non-white	1.03	0.48-2.19	.94			
Age (continuous)	1.02	1.01-1.03	<.001	1.02	1.01-1.03	<.001
Site melanoma						
Head and neck	Reference			Reference		
Trunk	1.11	0.68-1.81	.69	1.12	0.64-1.93	.70
Upper limbs/shoulder	1.29	0.73-2.26	.38	1.31	0.78-2.21	.30
Lower limbs/hip	0.66	0.34-1.28	.22	1.39	0.77-2.49	.27
Other	0.65	0.42-0.99	.05	0.68	0.34-1.38	.28
Histology						
SSM	Reference			Reference		
NM	0.64	0.33-1.23	.18	0.56	1.23-0.61	.56
LM	0.68	0.09-5.12	.71	0.49	0.41-1.52	.49
ALM	0.94	0.27-3.21	.92	0.73	0.10-5.61	.77
Melanoma, NOS	0.48	0.29-0.81	.006	1.47	0.40-5.43	.56
Other	0.70	0.36-1.39	.31	0.81	0.49-1.36	.43
Initial treatment						
Surgical treatment						
Yes	Reference			Reference		
No	0.60	0.45-0.80	<.001	1.34	0.85-2.09	.21
Radiation						
Yes	Reference			Reference		
No	1.74	1.22-2.47	.002	0.71	0.50-1.03	.07
Chemotherapy						
Yes	Reference			Reference		
No	1.55	1.10-2.18	.01	1.21	0.85-1.72	.30

[a]CI, confidence interval.

© 2020 Deng W et al. *JAMA Network Open*.

eTable 2. SIRs of all SPM sites after initial melanoma diagnosis between pre-ICIs and post-ICIs.

	2005-2010			2011-2016	
SPM sites	O	SIR (95% CI)		O	SIR (95% CI)
All Sites	107	1.65 (1.35-2)[1]		83	1.98 (1.57-2.45)[1]
All Sites excluding melanoma	77	1.26 (0.99-1.57)		56	1.42 (1.08-1.85)[1]
All Solid Tumors	89	1.56 (1.25-1.92)[1]		73	1.98 (1.55-2.49)[1]
Oral Cavity and Pharynx	1	0.57 (0.01-3.17)		1	0.8 (0.02-4.44)
Lip	0	0		0	0
Tongue	0	0		1	2.47 (0.06-13.79)
Salivary Gland	0	0		0	0
Floor of Mouth, and Gum and Other Mouth	1	3.2 (0.08-17.81)		0	0
Floor of Mouth	0	0		0	0
Gum and Other Mouth	1	4.43 (0.11-24.66)		0	0
Tonsil	0	0		0	0
Pharynx	0	0		0	0
Nasopharynx	0	0		0	0
Oropharynx	0	0		0	0
Hypopharynx	0	0		0	0
Other Oral Cavity and Pharynx	0	0		0	0
Digestive System	10	0.82 (0.39-1.51)		12	1.51 (0.78-2.63)
Esophagus	0	0		1	1.81 (0.05-10.09)
Stomach	3	2.85 (0.59-8.33)		2	2.89 (0.35-10.44)
Small Intestine	1	3.24 (0.08-18.04)		2	9.23 (1.12-33.35)[1]
Colon, Rectum and Anus	4	0.64 (0.17-1.64)		5	1.3 (0.42-3.04)
Colon and Rectum	4	0.66 (0.18-1.7)		4	1.09 (0.3-2.78)
Colon excluding Rectum	3	0.69 (0.14-2.02)		1	0.38 (0.01-2.13)
Cecum	0	0		1	1.76 (0.04-9.81)
Appendix	0	0		0	0
Ascending Colon	1	1.17 (0.03-6.5)		0	0
Hepatic Flexure	0	0		0	0
Transverse Colon	0	0		0	0
Splenic Flexure	0	0		0	0
Descending Colon	0	0		0	0
Sigmoid Colon	2	1.79 (0.22-6.47)		0	0
Large Intestine, NOS	0	0		0	0
Rectum and Rectosigmoid Junction	1	0.59 (0.01-3.28)		3	2.82 (0.58-8.25)
Rectosigmoid Junction	1	2.32 (0.06-12.94)		0	0
Rectum	0	0		3	3.69 (0.76-10.79)
Anus, Anal Canal and Anorectum	0	0		1	6.43 (0.16-35.83)
Rectum, Rectosig Junct, Anus, Anal Canal and Anorectum	1	0.52 (0.01-2.91)		4	3.28 (0.89-8.4)
	2005-2010			2011-2016	

© 2020 Deng W et al. *JAMA Network Open*.

SPM sites	O	SIR (95% CI)		O	SIR (95% CI)
Liver, Gallbladder, Intrahep Bile Duct and Other Biliary	1	0.59 (0.02-3.31)		1	0.83 (0.02-4.61)
Liver	0	0		0	0
Gallbladder	0	0		0	0
Intrahep and Extrahep Bile Ducts, and Other Biliary	1	2.37 (0.06-13.23)		1	3.21 (0.08-17.91)
Intrahepatic Bile Duct	0	0		1	8.84 (0.22-49.24)
Other Biliary	1	3.46 (0.09-19.25)		0	0
Pancreas	1	0.52 (0.01-2.92)		1	0.76 (0.02-4.22)
Retroperitoneum	0	0		0	0
Peritoneum, Omentum and Mesentery	0	0		0	0
Other Digestive Organs	0	0		0	0
Respiratory System	18	1.80 (1.06-2.84)[1]		10	1.59 (0.76-2.93)
Nose, Nasal Cavity and Middle Ear	0	0		0	0
Larynx	0	0		1	2.75 (0.07-15.33)
Pleura	0	0		0	0 (0-1233.33)
Lung, Bronchus, Trachea, Mediastinum and Other Resp Org	18	1.92 (1.14-3.04)[1]		9	1.54 (0.7-2.92)
Lung and Bronchus	18	1.93 (1.14-3.05)[1]		9	1.54 (0.71-2.93)
Trachea	0	0		0	0
Mediastinum and Other Respiratory Organs	0	0		0	0
Bones and Joints	1	14.9 (0.38-83.01)		0	0
Soft Tissue including Heart	1	2.57 (0.06-14.29)		2	7.33 (0.89-26.49)
Skin excluding Basal and Squamous	30	7.71 (5.2-11.01)[1]		29	9.93 (6.65-14.26)[1]
Melanoma of the Skin	30	8.45 (5.7-12.07)[1]		27	10.11 (6.66-14.71)[1]
Other Non-Epithelial Skin	0	0		2	7.98 (0.97-28.81)
Breast	6	1.1 (0.41-2.4)		5	1.51 (0.49-3.53)
Female Breast	6	1.13 (0.41-2.45)		5	1.55 (0.5-3.61)
Male Breast	0	0		0	0
Female Genital System	1	0.48 (0.01-2.7)		2	1.56 (0.19-5.63)
Cervix Uteri	1	5.31 (0.13-29.56)		0	0
Corpus and Uterus, NOS	0	0		2	2.74 (0.33-9.9)
Corpus Uteri	0	0		2	2.83 (0.34-10.21)
Uterus, NOS	0	0		0	0
Ovary	0	0		0	0
Vagina	0	0		0	0
Vulva	0	0		0	0
Other Female Genital Organs	0	0		0	0
Male Genital System	8	0.62 (0.27-1.22)		7	0.9 (0.36-1.86)
Prostate	8	0.63 (0.27-1.24)		7	0.92 (0.37-1.89)
Testis	0	0		0	0
	2005-2010			2011-2016	

© 2020 Deng W et al. *JAMA Network Open*.

SPM sites	O	SIR (95% CI)		O	SIR (95% CI)
Penis	0	0		0	0
Other Male Genital Organs	0	0		0	0
Urinary System	10	1.51 (0.72-2.78)		5	1.1 (0.36-2.56)
Urinary Bladder	3	0.73 (0.15-2.13)		1	0.35 (0.01-1.97)
Kidney and Renal Pelvis	6	2.58 (0.95-5.61)		4	2.49 (0.68-6.37)
Renal Pelvis, Ureter and Other Urinary Organs	1	3.04 (0.08-16.93)		0	0
Kidney	6	2.77 (1.02-6.03)[1]		4	2.66 (0.73-6.82)
Renal Pelvis	0	0		0	0
Ureter	1	9.64 (0.24-53.68)		0	0
Other Urinary Organs	0	0		0	0
Eye and Orbit	0	0		0	0
Eye and Orbit - Non-Melanoma	0	0		0	0
Eye and Orbit - Melanoma	0	0		0	0
Brain and Other Nervous System	1	1.38 (0.03-7.7)		1	2.08 (0.05-11.61)
Brain	1	1.44 (0.04-8.02)		1	2.16 (0.05-12.05)
Cranial Nerves Other Nervous System	0	0		0	0
Endocrine System	2	2 (0.24-7.22)		0	0
Thyroid	2	2.13 (0.26-7.7)		0	0
Thymus, Adrenal Gland and Other Endocrine	0	0		0	0
Thymus	0	0		0	0
Adrenal Gland	0	0		0	0
Other Endocrine	0	0		0	0
All Lymphatic and Hematopoietic Diseases	17	2.86 (1.67-4.58)[1]		7	1.72 (0.69-3.55)
Lymphoma	4	1.31 (0.36-3.36)		3	1.46 (0.3-4.28)
Hodgkin Lymphoma	0	0		0	0
Hodgkin - Nodal	0	0		0	0
Hodgkin - Extranodal	0	0		0	0
Non-Hodgkin Lymphoma	4	1.39 (0.38-3.57)		3	1.55 (0.32-4.52)
NHL - Nodal	3	1.56 (0.32-4.55)		2	1.56 (0.19-5.65)
NHL - Extranodal	1	1.06 (0.03-5.9)		1	1.51 (0.04-8.42)
Myeloma	7	7.29 (2.93-15.02)[1]		4	5.90 (1.61-15.1)[1]
Leukemia	6	3.11 (1.14-6.76)[1]		0	0
Lymphocytic Leukemia	4	3.98 (1.09-10.2)[1]		0	0
Acute Lymphocytic Leukemia	0	0		0	0
Chronic Lymphocytic Leukemia	4	4.57 (1.24-11.7)[1]		0	0
Other Lymphocytic Leukemia	0	0		0	0
Non-Lymphocytic Leukemia	2	2.16 (0.26-7.79)		0	0
Acute Non-Lymphocytic Leukemia (ANLL)	1	1.62 (0.04-9.05)		0	0
Myeloid and Monocytic Leukemia	1	1.18 (0.03-6.6)		0	0
	2005-2010			2011-2016	

© 2020 Deng W et al. *JAMA Network Open*.

SPM sites	O	SIR (95% CI)		O	SIR (95% CI)
Acute Myeloid Leukemia	1	1.81 (0.05-10.09)		0	0
Acute Monocytic Leukemia	0	0		0	0
Chronic Myeloid Leukemia	0	0		0	0
Other Myeloid/Monocytic Leukemia	0	0		0	0
Other Leukemia	1	11.94 (0.3-66.51)		0	0
Other Acute Leukemia	0	0		0	0
Aleukemic, Subleukemic and NOS	1	19.85 (0.5-110.59)		0	0
Mesothelioma	0	0		1	7.47 (0.19-41.61)
Kaposi Sarcoma	0	0		0	0
Miscellaneous	1	0.75 (0.02-4.17)		1	1.17 (0.03-6.52)

[1]Indicateds a statistically significant value; O: observed number; SIR: Standardized incidence ratio.

© 2020 Deng W et al. *JAMA Network Open*.

eTable 3. SIRs of selected SPMs sites after initial melanoma diagnosis between males and females.

	Male			Female	
SPM sites	O	SIR (95% CI)		O	SIR (95% CI)
All Sites	138	1.80 (1.51-2.12)[1]		52	1.73 (1.3-2.27)[1]
All Sites excluding melanoma	96	1.34 (1.08-1.63)[1]		37	1.29 (0.91-1.77)
All Solid Tumors	115	1.71 (1.41-2.05)[1]		47	1.76 (1.3-2.34)[1]
Oral Cavity and Pharynx	2	0.79 (0.1-2.85)[1]		0	0
Lip	0	0		0	0
Tongue	1	1.24 (0.03-6.91)[1]		0	0
Salivary Gland	0	0		0	0
Floor of Mouth, and Gum and Other Mouth	1	2.58 (0.07-14.37)		0	0
Floor of Mouth	0	0		0	0
Gum and Other Mouth	1	3.65 (0.09-20.34)		0	0
Tonsil	0	0		0	0
Pharynx	0	0		0	0
Nasopharynx	0	0		0	0
Oropharynx	0	0		0	0
Hypopharynx	0	0		0	0
Other Oral Cavity and Pharynx	0	0		0	0
Digestive System	15	1.02 (0.57-1.68)		7	1.29 (0.52-2.66)
Esophagus	1	0.82 (0.02-4.59)		0	0
Stomach	4	2.92 (0.8-7.48)		1	2.67 (0.07-14.85)
Small Intestine	2	5.26 (0.64-19.01)		1	6.88 (0.17-38.31)
Colon, Rectum and Anus	5	0.71 (0.23-1.65)		4	1.32 (0.36-3.39)
Colon and Rectum	5	0.73 (0.24-1.7)		3	1.05 (0.22-3.07)
Colon excluding Rectum	2	0.42 (0.05-1.51)		2	0.92 (0.11-3.31)
Cecum	0	0		1	1.82 (0.05-10.16)
Appendix	0	0		0	0
Ascending Colon	1	1.1 (0.03-6.15)		0	0
Hepatic Flexure	0	0		0	0
Transverse Colon	0	0		0	0
Splenic Flexure	0	0		0	0
Descending Colon	0	0		0	0
Sigmoid Colon	1	0.76 (0.02-4.26)		1	2.18 (0.06-12.16)
Large Intestine, NOS	0	0		0	0
Rectum and Rectosigmoid Junction	3	1.44 (0.3-4.2)		1	1.48 (0.04-8.27)
Rectosigmoid Junction	0	0		1	5.7 (0.14-31.74)
Rectum	3	1.9 (0.39-5.54)		0	0
Anus, Anal Canal and Anorectum	0	0		1	5.96 (0.15-33.18)
Rectum, Rectosig Junct, Anus, Anal Canal and Anorectum	3	1.31 (0.27-3.83)		2	2.38 (0.29-8.58)

© 2020 Deng W et al. *JAMA Network Open*.

	Male			Female	
SPM sites	O	SIR (95% CI)		O	SIR (95% CI)
Liver, Gallbladder, Intrahep Bile Duct and Other Biliary	2	0.87 (0.11-3.15)		0	0
Liver	0	0		0	0
Gallbladder	0	0		0	0
Intrahep and Extrahep Bile Ducts, and Other Biliary	2	3.8 (0.46-13.72)		0	0
Intrahepatic Bile Duct	1	5.83 (0.15-32.46)		0	0
Other Biliary	1	2.82 (0.07-15.69)		0	0
Pancreas	1	0.44 (0.01-2.47)		1	1.03 (0.03-5.72)
Retroperitoneum	0	0		0	0
Peritoneum, Omentum and Mesentery	0	0		0	0
Other Digestive Organs	0	0		0	0
Respiratory System	23	1.95 (1.24-2.92)[1]		5	1.11 (0.36-2.59)
Nose, Nasal Cavity and Middle Ear	0	0		0	0
Larynx	1	1.18 (0.03-6.58)		0	0
Pleura	0	0		0	0
Lung, Bronchus, Trachea, Mediastinum and Other Resp Org	22	2.03 (1.27-3.08)[1]		5	1.14 (0.37-2.66)
Lung and Bronchus	22	2.04 (1.28-3.08)[1]		5	1.14 (0.37-2.67)
Trachea	0	0		0	0
Mediastinum and Other Respiratory Organs	0	0		0	0
Bones and Joints	1	12.07 (0.31-67.24)		0	0
Soft Tissue including Heart	2	4 (0.48-14.44)		1	6.17 (0.16-34.38)
Skin excluding Basal and Squamous	44	8.06 (5.86-10.82)[1]		15	11.10 (6.21-18.31)[1]
Melanoma of the Skin	42	8.42 (6.07-11.39)[1]		15	12.16 (6.8-20.05)[1]
Other Non-Epithelial Skin	2	4.23 (0.51-15.27)		0	0
Breast	0	0		11	1.29 (0.64-2.3)
Female Breast	0	0		11	1.29 (0.64-2.3)
Male Breast	0	0		0	0
Female Genital System	0	0		3	0.9 (0.18-2.62)
Cervix Uteri	0	0		1	3.33 (0.08-18.53)
Corpus and Uterus, NOS	0	0		2	1.07 (0.13-3.88)
Corpus Uteri	0	0		2	1.11 (0.13-4)
Uterus, NOS	0	0		0	0
Ovary	0	0		0	0
Vagina	0	0		0	0
Vulva	0	0		0	0
Other Female Genital Organs	0	0		0	0
Male Genital System	15	0.73 (0.41-1.2)		0	0
Prostate	15	0.74 (0.41-1.22)		0	0
Testis	0	0		0	0

© 2020 Deng W et al. *JAMA Network Open*.

	Male			Female	
SPM sites	O	SIR (95% CI)		O	SIR (95% CI)
Penis	0	0		0	0
Other Male Genital Organs	0	0		0	0
Urinary System	11	1.15 (0.58-2.07)		4	2.42 (0.66-6.2)
Urinary Bladder	4	0.65 (0.18-1.67)		0	0
Kidney and Renal Pelvis	6	1.91 (0.7-4.16)		4	5.03 (1.37-12.87)[1]
Renal Pelvis, Ureter and Other Urinary Organs	1	2.27 (0.06-12.64)		0	0
Kidney	6	2.04 (0.75-4.45)		4	5.47 (1.49-14.02)[1]
Renal Pelvis	0	0		0	0
Ureter	1	7.23 (0.18-40.27)		0	0
Other Urinary Organs	0	0		0	0
Eye and Orbit	0	0		0	0
Eye and Orbit - Non-Melanoma	0	0		0	0
Eye and Orbit - Melanoma	0	0		0	0
Brain and Other Nervous System	1	1.12 (0.03-6.27)		1	3.18 (0.08-17.72)
Brain	1	1.17 (0.03-6.49)		1	3.34 (0.08-18.61)
Cranial Nerves Other Nervous System	0	0		0	0
Endocrine System	2	2.39 (0.29-8.65)		0	0
Thyroid	2	2.62 (0.32-9.47)		0	0
Thymus, Adrenal Gland and Other Endocrine	0	0		0	0
Thymus	0	0		0	0
Adrenal Gland	0	0		0	0
Other Endocrine	0	0		0	0
All Lymphatic and Hematopoietic Diseases	20	2.68 (1.64-4.15)[1]		4	1.57 (0.43-4.01)
Lymphoma	7	1.88 (0.76-3.87)		0	0
Hodgkin Lymphoma	0	0		0	0
Hodgkin - Nodal	0	0		0	0
Hodgkin - Extranodal	0	0		0	0
Non-Hodgkin Lymphoma	7	1.99 (0.8-4.11)		0	0
NHL - Nodal	5	2.13 (0.69-4.97)		0	0
NHL - Extranodal	2	1.72 (0.21-6.21)		0	0
Myeloma	8	6.49 (2.8-12.8)[1]		3	7.38 (1.52-21.57)[1]
Leukemia	5	2 (0.65-4.67)		1	1.3 (0.03-7.23)
Lymphocytic Leukemia	3	2.29 (0.47-6.7)		1	2.68 (0.07-14.95)
Acute Lymphocytic Leukemia	0	0		0	0
Chronic Lymphocytic Leukemia	3	2.63 (0.54-7.7)		1	3.06 (0.08-17.05)
Other Lymphocytic Leukemia	0	0		0	0
Non-Lymphocytic Leukemia	2	1.68 (0.2-6.08)		0	0
Acute Non-Lymphocytic Leukemia (ANLL)	1	1.26 (0.03-7.03)		0	0
Myeloid and Monocytic Leukemia	1	0.91 (0.02-5.08)		0	0

© 2020 Deng W et al. *JAMA Network Open*.

	Male			Female	
SPM sites		SIR (95% CI)			SIR (95% CI)
Acute Myeloid Leukemia	1	1.4 (0.04-7.78)		0	0
Acute Monocytic Leukemia	0	0		0	0
Chronic Myeloid Leukemia	0	0		0	0
Other Myeloid/Monocytic Leukemia	0	0		0	0
Other Leukemia	1	10.78 (0.27-60.08)		0	0
Other Acute Leukemia	0	0		0	0
Aleukemic, Subleukemic and NOS	1	18.35 (0.46-102.23)		0	0
Mesothelioma	1	3.34 (0.08-18.61)		0	0
Kaposi Sarcoma	0	0		0	0
Miscellaneous	1	0.65 (0.02-3.64)		1	1.52 (0.04-8.47)

[1]Indicateds a statistically significant value; O: observed number; SIR: Standardized incidence ratio.

© 2020 Deng W et al. *JAMA Network Open*.
